# Elevated ovarian pentraxin 3 in polycystic ovary syndrome

**DOI:** 10.1007/s10815-021-02105-4

**Published:** 2021-02-16

**Authors:** Jiexue Pan, Chengliang Zhou, Zhiyang Zhou, Zuwei Yang, Tiantian Dai, Hefeng Huang, Li Jin

**Affiliations:** 1 The International Peace Maternal and Child Health Hospital, School of Medicine, Shanghai Jiao Tong University, Shanghai, 200030 China; 2 Obstetrics & Gynecology Hospital, Institute of Reproduction and Development, Fudan University, Shanghai, 200011 China; 3 Shanghai Key Laboratory of Embryo Original Diseases, Shanghai, 200030 China; 4 Shanghai Ji Ai Genetics and IVF Institute, Obstetrics and Gynecology Hospital, Fudan University, Shanghai, 200011 China; 5 The Key Laboratory of Reproductive Genetics, Ministry of Education (Zhejiang University), Zhejiang, 310006 Hangzhou China

**Keywords:** Polycystic ovary syndrome, PTX3, Hyperandrogenism, Follicular fluid, Innate immunology

## Abstract

**Purpose:**

Pentraxin 3 (PTX3) plays a crucial role in cumulus expansion and fertilization. The ovarian PTX3 level in polycystic ovary syndrome (PCOS) remains uncertain. In the present study, we investigated the follicular PTX3 levels and found the influence of reproductive hormones on ovarian PTX3 concentration.

**Methods:**

This study was based on 204 healthy-weight women (102 PCOS and 102 normal ovulating subjects) undergoing in vitro fertilization (IVF). Follicular fluid (FF) was collected during oocyte retrieval. The PTX3 levels and other hormone levels in FF samples were analyzed by enzyme-linked immunosorbent assay (ELISA).

**Results:**

The PTX3 level in the follicle was significantly higher in the healthy-weight PCOS women than controls. Positive correlations were found between ovarian PTX3 level and the existence of PCOS, cycle length, basal LH to FSH ratio and TT in serum, antral follicle count, and ovarian insulin and androgen level, and inverse correlations with the basal serum PRL and ovarian SHBG. In multivariant linear regression analysis, the presence of PCOS diagnosis, participants’ basal LH to FSH ratio, and ovarian androstenedione level were the main predictors of ovarian PTX3 level among the enrolled subjects.

**Conclusion:**

Elevated ovarian PTX3 level supports the low-grade chronic inflammatory state in the follicles of PCOS. The existence of PCOS, disturbed pituitary gland, and ovarian hyperandrogenism might also be related to this state of low-grade chronic inflammation and could be a subject of further study.

## Introduction

Polycystic ovary syndrome (PCOS) is one of the most common endocrinopathies, afflicting females of reproductive age, with an estimated prevalence of 5–19.9% according to different diagnostic criteria [1, 2]. It is characterized by hyperandrogenism, oligo-/anovulation, and polycystic ovarian morphology. Metabolic dysfunction in PCOS includes increased incidence of insulin resistance, hyperinsulinemia, dyslipidemia, and cardiovascular diseases. Detailed pathogenesis of PCOS remains to be illustrated. Emerging data suggest that PCOS is a multifactorial disease, and genetic [3], epigenetic [4], and environmental [5] factors could determine individual susceptibility of this condition.

Accumulating evidence suggests that PCOS is a chronic inflammatory disease. The ovaries of women with PCOS exhibit inflammation and fibrosis [6]; the peripheral blood of women with PCOS has reduced numbers of anti-inflammatory regulatory T cells and elevated serum levels of autoantibodies [7], and recent study also indicated the pathogenic role for CD19^+^ B cells in the development of PCOS [8].

Humoral innate immune molecule pentraxin 3 (PTX3) that belongs to the same superfamily of acute reactants as C-reactive protein (CRP) is a member of the long pentraxin family and has multifunctional properties for its capacity to interact with different types of ligands. In particular, PTX3 plays a non-redundant role in innate immunity by opsonizing selected pathogens [9] and in female fertility [10, 11]. Recent research demonstrated that PTX3 in the circulation is associated with PCOS [12–14], but its role in PCOS is so far inconclusive. Unlike the short pentraxin, PTX3 is produced in the local site of the inflammation, including follicle cells [4], and it is essential for the organization of cumulus oophorus extracellular matrix and in vivo fertilization [15, 16]. The PTX3 level in the follicular fluid has not been tested in PCOS women, yet. In this study, we aimed to assess whether the ovarian PTX3 level is altered in PCOS women and analyze the relationships between follicular PTX3 level and clinical and hormonal features.

## Materials and methods

### Patient selection and sample collection

The study protocol conforms to the ethical guidelines of the 1975 Declaration of Helsinki as reflected in a priori approval by the Ethics Committee of the International Peace Maternity and Child Health Hospital, School of Medicine, Shanghai Jiao Tong University. All participants provided written informed consent after full explanation of the purpose and nature of all procedures used. One hundred two PCOS patients, diagnosed according to the Rotterdam Consensus (European Society for Human Reproduction and Embryology/American Society for Reproductive Medicine criteria) [6], and one hundred two infertile women with tubal blockage (serving as controls) seeking in vitro fertilization (IVF) treatment were recruited. The control women met the following inclusion criteria: (1) normal ovarian response; (2) menstrual cycle length range between 26 and 33 days; (3) no structural abnormalities of the uterus and ovary was found by vaginal ultrasound and/or laparoscopy; (4) no diseases affecting gonadotropin and sex steroid secretion, clearance, or excretion; (5) no signs of hyperandrogenism; and (6) no polycystic ovary morphology or other morphological abnormalities. And all enrolled subjects met the following inclusion criteria: (1) age between 22 and 37; (2) body mass index (BMI) between 18.5 and 25; (3) both ovaries present; (4) only the subjects undergoing GnRH long agonist protocol or GnRH antagonist protocol were selected; (5) all the participants undergoing intracytoplasmic sperm injection (ICSI) or whose spouse had male infertility factors were excluded. The FF samples were carefully collected from the first aspiration follicle as previously described [4], and only FF samples from one follicle which did not contain any visible blood contamination were used in this study. The FF samples were immediately centrifuged, and the supernatants were stored at − 80 °C until further analysis.

### Measurement of hormones

The levels of day 3 serum hormones were measured by chemiluminescence immunoassay (CLIA) in the clinical laboratory of International Peace Maternity and Child Health Hospital, School of Medicine, Shanghai Jiao Tong University. The hormone levels in FFs were detected by enzyme-linked immunosorbent assay (ELISA), including androstenedione (Abcam, Cambridge, UK), total testosterone (TT) (Abcam, Cambridge, UK), sex hormone-binding globulin (SHBG, R&D Systems, Inc. Minneapolis, MN, USA), and insulin (R&D Systems, Inc. Minneapolis, MN, USA). Free androgen index (FAI) was calculated as the ratio of TT (nmol/L)/SHBG (nmol/L) multiplied by 100. The values in FFs were from the individual follicle from each subject.

### Measurement PTX3 levels in FF

The concentration of PTX3 in FF was determined using an ELISA kit (R&D Systems, Inc. Minneapolis, MN, USA), according to the manufacturer’s protocol. This assay is a quantitative sandwich enzyme immunoassay technique. The intra- and interassay coefficients of variations were 3.7% and 7.3%, respectively. The assay was performed using FF that had been stored frozen at − 80 °C and the PTX3 levels were all measured at the same time using reagents with the same lot numbers to reduce the measurement variability.

### Statistical analysis

Statistical analysis is performed using the software SPSS version 24.0 software (SPSS Inc., Chicago, IL, USA). Differences in the demographic and clinical characteristics among the subjects were evaluated by *χ*^2^, *t* tests, or non-parametric Mann–Whitney *U* test according to whether the variables were categorical or continuous. The PTX3 distribution was non-normal, so the non-parametric Mann–Whitney *U* test was applied. The correlations between FF PTX3 levels and other components participating in our study were analyzed using Pearson’s correlation. Multivariate analysis was performed using backward stepwise logistic regression analysis. All tests were two-sided and *P* < 0.05 was considered statistically significant.

## Results

### Patient demographic data and clinical features

Demographic data and baseline clinical characteristics of cases and controls are shown in Table 1. Enrolled PCOS women and control ones were of comparable age and duration of infertility. The GnRHant protocol was more common among the enrolled subjects. Significant differences between the two groups (*P* < 0.05) could be found in menstrual cycle length, parity, body mass index (BMI), antral follicle count (AFC), serum luteinizing hormone (LH)/follicle-stimulating hormone (FSH) ratio, total testosterone (TT), dehydroepiandrosterone-sulfate (DHEA-S), prolactin (PRL), and estradiol (E_2_) levels on the third day of menstrual cycle. All these values mentioned above were greater for PCOS group than those in controls except for serum PRL level, which is lower in PCOS women. The PCOS women were more likely to suffer from primary infertility. The fertilization rate of the enrolled PCOS women was significantly lower than that of the controls. The average follicular diameter used for the analysis of FF is 16 mm, and the minimum follicular diameter used to obtain FF is no less than 14 mm. Furthermore, the concentrations of androstenedione, TT, free androgen index (FAI), and insulin in the follicular fluid (FF) were significantly higher in the PCOS group, whereas the sex hormone-binding globulin (SHBG) level was lower.

**Table 1:** Demographic data and clinical characteristics of IVF patients, between control and PCOS groups

Items	Control (*n* = 102)	PCOS (*n* = 102)	*P* value
Age	29.98 ± 0.36	29.17 ± 0.35	0.108
Body mass index (kg/m^2^)	21.39 ± 0.21	22.02 ± 0.21	0.040
Cycle length (days)	28.95 ± 0.17	67.56 ± 4.04	< 0.001
Duration of infertility (years)	4.27 ± 0.31	4.20 ± 0.38	0.927
Parity, *n* (%)			< 0.001
No previous pregnancy	40 (39.22)	66 (64.71)	
Previous pregnancy	40 (60.78)	36 (35.29)	
Day 3 LH/FSH	0.77 ± 0.02	1.35 ± 0.08	< 0.001
Day 3 TT (nmol/L)	0.79 ± 0.03	2.00 ± 0.08	< 0.001
Day 3 DHEA-S (umol/L)	6.68 ± 0.21	7.95 ± 0.31	0.001
Day 3 PRL (ng/ml)	15.53 ± 0.57	13.81 ± 0.55	0.031
Day 3 E_2_ (pmol/L)	135.03 ± 4.06	159.24 ± 6.95	0.003
AFC	10.82 ± 0.23	24.59 ± 0.26	< 0.001
Protocols, *n* (%)			0.203
GnRHa long	22 (21.6)	15 (14.7)	
GnRHant	80 (78.4)	87 (85.3)	
Fertilization rate	72.84 ± 2.16	61.38 ± 2.21	< 0.001
Androstenedione (ng/ml) in FF	6.27 ± 0.34	8.94 ± 1.01	0.013
TT (ng/ml) in FF	5.22± 0.22	9.17 ± 0.55	< 0.001
SHBG (nmol/L) in FF	57.17 ± 1.10	51.86 ± 1.32	0.002
FAI in FF	32.50 ± 1.39	68.20 ± 5.96	< 0.001
Insulin (mU/L) in FF	38.80 ± 1.66	50.43 ± 2.10	< 0.001
PTX3 (ng/ml) in FF	8.60 (6.48–13.52)	15.59 (11.92–23.10)	< 0.001

Day 3, the third day of spontaneous menstrual cycle; *LH*, luteinizing hormone; *FSH*, follicle-stimulating hormone; *TT*, total testosterone; *DHEA-S*, dehydroepiandrosterone-sulfate; *PRL*, prolactin; *E*_*2*_, estradiol; *AFC*, antral follicle count; *GnRHa*, gonadotropin-releasing hormone agonist; *GnRHant*, gonadotropin-releasing hormone antagonist; *Gn*, gonadotrophin; *HCG*, human chorionic gonadotropin; *FF*, follicular fluid; *SHBG*, sex hormone-binding globulin; *FAI*, free androgen index; *PTX3*, pentraxin 3. Values are presented as mean ± SE or median (lower quartile–upper quartile). Significance was determined by unpaired Student’s *t* test, *χ*^2^, or non-parametric Mann–Whitney *U* test

### Ovarian PTX3 levels in PCOS women and controls

**Fig. 1:**
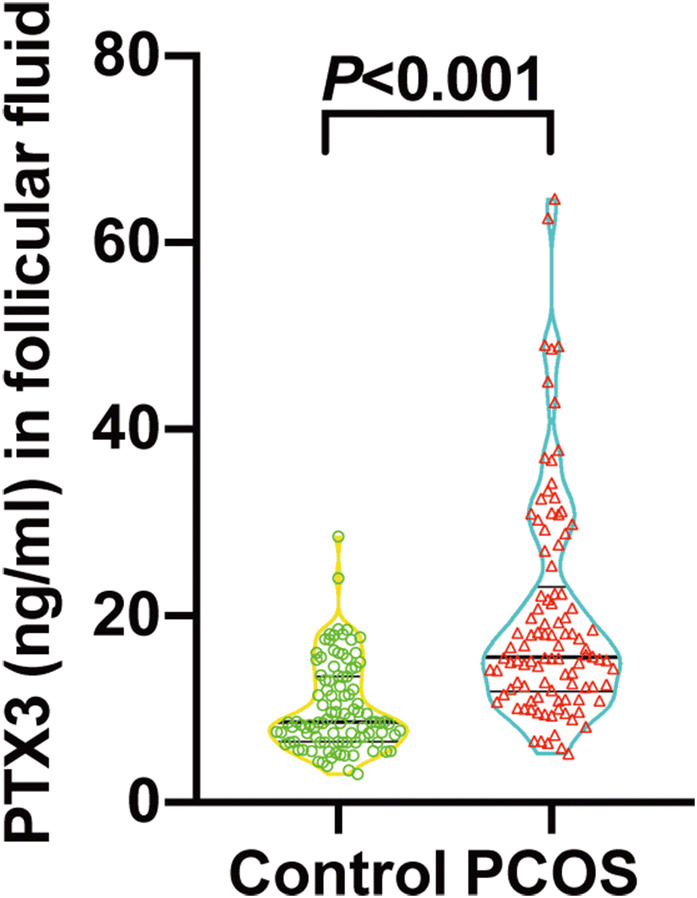
Ovarian PTX3 levels among normal ovulating women and PCOS subjects. The bottom and top of each box indicate the 25th and 75th percentiles, the line through the middle of each box represents the median. *P* value is determined by non-parametric Mann–Whitney *U* test

As shown in Table 1 and Fig. 1, the ovarian PTX3 level was 8.60 (95% CI: 4.42–18.05) ng/ml and 15.59 (95% CI: 6.64–48.13) ng/ml in control and PCOS women, respectively. We observed a significant elevation of 94% in the mean ovarian PTX3 level from PCOS women (19.76 ± 1.17 ng/ml) compared with controls (10.18 ± 0.47 ng/ml) (*P* < 0.001).

### Associations of enrolled parameters with ovarian PTX3 levels in PCOS

Table 2 shows the univariated relationships between the ovarian PTX3 level and several anthropometric, endocrine, and metabolic parameters. In the whole sample of PCOS and control subjects, correlation analysis revealed that the PTX3 level in FF was positively associated with the existence of PCOS (*R* = 0.534, *P* < 0.001), cycle length (*R* = 0.425, *P* < 0.001), basal LH to FSH ratio (*R* = 0.398, *P* < 0.001), and TT (*R* = 0.475, *P* < 0.001) in serum, AFC (*R* = 0.467, *P* < 0.001), ovarian insulin level (*R* = 0.179, *P* = 0.010), and ovarian hyperandrogenism (*R* = 0.616 for androstenedione; *R* = 0.664 for TT; *R* = 0.668 for FAI, all *P* < 0.001). And it was inversely associated with the basal serum PRL (*R* = − 0.180, *P* = 0.010) and ovarian SHBG (*R* = − 0.156, *P* = 0.026) (Table 2). However, no relationships were observed between the ovarian PTX3 level and age, BMI, duration of infertility, parity, basal serum DHEA-S and E_2_ levels, COH parameters, and fertilization rate.

**Table 2:** Correlations of follicular PTX3 with each parameter

Items	*R*	*P* value
Existence of PCOS	0.534	< 0.001
Age	− 0.122	0.083
Body Mass Index (kg/m^2^)	0.101	0.151
Cycle length (days)	0.425	< 0.001
Duration of infertility (years)	− 0.067	0.343
Parity	− 0.103	0.141
Day 3 LH/FSH	0.398	< 0.001
Day 3 TT	0.475	< 0.001
Day 3 DHEA-S	0.100	0.154
Day 3 PRL	− 0.180	0.010
Day 3 E_2_	0.094	0.182
AFC	0.467	< 0.001
Protocols	0.045	0.520
Induction length (days)	0.016	0.816
E_2_ (pmol/L) on HGC day	− 0.060	0.397
No. of retrieved oocytes	0.103	0.141
Fertilization rate	− 0.121	0.086
Androstenedione in FF	0.616	< 0.001
TT in FF	0.664	< 0.001
SHBG in FF	− 0.156	0.026
FAI in FF	0.668	< 0.001
Insulin in FF	0.179	0.010

Data are presented as Pearson correlation coefficients

The multivariable associations between follicular PTX3 with possible variables are displayed in Table 3. The PTX3 level in the ovary was pronouncedly associated with the existence of PCOS (95% CI = 3.704~8.711), serum LH to FSH ratio on the third day of menstrual cycles (95% CI = 1.704~4.898), and ovarian androstenedione (95% CI = 0.600~0.846). In a multivariable regression model, the associations of ovarian PTX3 with menstrual cycle length, basal PRL, TT, AFC, and ovarian TT, FAI, SHBG, and insulin levels were no longer significant.

**Table 3:** Stepwise multiple regression analysis between follicular PTX3 levels and each parameter

Variable	Unstandardized coefficients		Standardized coefficients	*P* value	(95% CI)
	B	Std. Error	Beta		
Constant	− 2.253	1.521		0.140	(− 5.252~0.747)
Existence of PCOS	6.207	1.269	0.305	< 0.001	(3.704~8.711)
Day 3 LH/FSH	3.301	0.810	0.231	< 0.001	(1.704~4.898)
Androstenedione in FF	0.723	0.0762	0.539	< 0.001	(0.600~0.846)
Adjusted *R*^2^	0.566				
*P* value of the model	< 0.001				
Std. error of the estimate	6.383				

These factors were adjusted in the multivariate regression analysis: menstrual cycle length, day3 PRL, day3 TT, AFC, TT in FF, FAI in FF, SHBG in FF, insulin in FF

## Discussion

In the present study, we found that the ovarian PTX3 is increased in non-obese PCOS women. In addition, the existence of PCOS, serum basal LH/FSH ratio, and high levels of ovarian androstenedione were strongly associated with the ovarian PTX3 level. To our knowledge, this study, for the first time, explored the ovarian PTX3 level in PCOS women and investigated the relationships between ovarian PTX3 level and other hormones.

Up to now, there were several studies investigating serum PTX3 level in PCOS, but the association between PTX3 and PCOS remains uncertain. Among these studies, serum PTX3 level in PCOS women was found to be lower [12, 13], higher [14, 17], or comparable [18] to that of non-PCOS women. In contrast to the short pentraxin, CRP, which is predominantly produced in the liver, PTX3 is synthesized in response to local inflammatory stimuli by a variety of cells, and ovarian follicular cells have innate immune capabilities that modulate their endocrine function [19]. Our previous study has found that the granulosa cells from PCOS women had higher expression of PTX3 than the non-PCOS women [4]. So it is of great significance to evaluate the PTX3 level in the ovarian environment. Mean PTX3 level in the follicle was 10.18 ± 0.47 ng/mL in the normal ovulating women, which is similar to the finding of Alessio et al. [20]. The mean concentration of follicular PTX3 in PCOS women was 19.76 ± 1.17 ng/mL, which was clearly higher compared to normal ovulating controls. Studies in mice have revealed that *Ptx3* mRNA expression starts 2 h after injection of hCG, peaks at 6 h, and declines thereafter [15], coinciding with matrix deposition by cumulus cells and cumulus expansion [21]. It might be hypothesized that the variability in time between hCG injection and ovum pick-up could account for elevated follicular PTX3 in PCOS women. But oocyte retrieval is usually performed at 36 h after hCG administration, so our results provide a basis for pursuing studies on the innate immunity in the pathogenesis of PCOS, and PXT3 might also have specific diagnostic and immunologic therapeutic value in PCOS.

We failed to find any significant association between fertilization rate and follicular concentration of PTX3. However, the fertilization rate here refers to the retrieved oocytes of one women, not the oocytes that come from the examined FF, so it is more meaningful to perform in vitro study to evaluate the effect of PTX3 on fertilization. There is a constant state of chronic low-grade inflammation in obesity and insulin resistance [22]. Samples from the non-obese women were selected in our study to minimize the influences of obesity and metabolic disturbances on inflammatory status. The uncertained association between plasma PTX3 level and metabolic disturbances has been discussed in several studies [13, 14]. Previous studies related to male subjects have reported inverse link between plasma PTX3 and fat mass [23], BMI [24], and waist circumference [24], while positive correlation between PTX3 levels and BMI as well as visceral obesity in PCOS was observed [14]. A recent study carried out by Katarzyna et al. also showed a positive correlation between circulating PTX3 levels and BMI values and fat percentage in women with PCOS, while the correlation was negative in non-PCOS women [17]. It was reported that PTX3 mRNA expression is upregulated in visceral adipose tissue in obesity, whereas the plasma PTX3 levels inversely correlated with insulin secretion [25]. The discrepancies of these studies are not easily explained. In our study, the enrolled PCOS women had higher BMI and ovarian insulin level than the control, but further association analysis indicated that ovarian PTX3 level was not correlated with BMI or ovarian insulin level among the enrolled subjects. Possible explanation of this finding is the concentration of BMI that varies from 18.5 to 25. Most PCOS subjects from other studies are overweight or obese with higher BMI or fat percentage and the enrolled subjects in our study compromised Chinese women with lower BMI values. Since PTX3 can be secreted by follicular cells and the existence of blood-follicle barrier that separates FF from blood capillaries [26], and we have excluded the obese women during the study, it is reasonable to have such disaccord with that in the circulation.

Hyperandrogenism, especially the excessive androgen production by the ovaries, is the core pathophysiologic feature of PCOS [4, 27]. The enrolled PCOS women in our study showed higher androgen levels both in the circulation and ovary. Ibrahim et al. had reported that hyperandrogenism might be related to the low-grade chronic inflammatory state in PCOS women [28]. Among the enrolled subjects, we found significantly positive correlations between ovarian PTX3 level and endocrine disturbances of this condition, including higher ovarian and circulating androgen levels. Further regression analysis revealed that the correlations of PTX3 level in follicle with menstrual cycle length, basal PRL, TT, AFC, and follicular TT, FAI, SHBG, and insulin were attenuated. Only the existence of PCOS, higher ratio of LH/FSH, and elevated follicular androstenedione could account for the higher PTX3 in the ovary.

The LH hypersecretion by the pituitary gland also induces ovary dysfunction and enhances hypersecretion of androgen in the theca cells in ovarian follicle [2], thus inducing hyperandrogenism both in the circulation and ovary. The remarkable female predominance of autoimmune diseases has suggested the immunosuppressive effects of androgens [29], while other investigators reported a direct proinflammatory effect of androgens on mononuclear cell [30, 31]. Despite a documented role of androgen as an immunoregulator with organ-specific manner, the potential role of hyperandrogenism on the immune response, especially innate immune response of ovary, has never been fully illustrated. PCOS is characterized by a chronic inflammatory state both in circulation [28, 32] and ovaries [33]. Ovary acts as the main androgen supplier in women, and is also an androgen-sensitive tissue [34]. Studies have indicated that androgen excess in vivo and in vitro induces a proinflammatory response in PCOS women [35, 36]. In our study, the univariable association analysis and multivariable regression analysis revealed remarkable positive associations between follicular PTX3 level and ovarian androstenedione concentration. The finding suggests that ovarian hyperandrogenism might be the causation of elevated PTX3 in the follicle. *Ptx3* expression in the follicle cells is regulated by growth differentiation factor 9, which is regulated by androgen/androgen receptor [37]. The RNA-seq data indicated the upregulation of PTX3 in follicle cells from PCOS women is associated with hyperandrogenism [4]. However, there is no available data on the direct effect of androgens on PTX3 production. Further studies are warranted to clarify the potential role of endocrine mediators in the development of higher PTX3 in the ovary.

There are several limitations and considerations with regard to the present study. All the enrolled subjects were undergoing assisted reproductive medicine and all the samples were collected after some drug administration. Secondly, the subjects enrolled in our study were non-obese, so it is worth further verification in both obese and non-obese subjects, or other ethnic groups should be undertaken with caution. What’s more, the enrolled samples were all from luteinized follicles. Therefore, it is of significance to further confirm our findings in unstimulated and non-luteinized follicular fluid, and also this can be further carried out in animal study.

## References

[1] Lizneva D, Suturina L, Walker W, Brakta S, Gavrilova-Jordan L, Ricardo A (2016). Criteria, prevalence, and phenotypes of polycystic ovary syndrome. Fertil Steril.

[2] Azziz R, Carmina E, Chen Z, Dunaif A, Laven JS, Legro RS, et al. Polycystic ovary syndrome. Nat Rev Dis Primers. 2016;2. 10.1038/nrdp.2016.57. 10.1038/nrdp.2016.57 27510637

[3] Azziz R (2016). PCOS in 2015: new insights into the genetics of polycystic ovary syndrome. Nat Rev Endocrinol.

[4] Pan J-X, Tan Y-J, Wang F-F, Hou N-N, Xiang Y-Q, Zhang J-Y (2018). Aberrant expression and DNA methylation of lipid metabolism genes in PCOS: a new insight into its pathogenesis. Clin Epigenetics.

[5] Palioura E, Diamanti-Kandarakis E (2015). Polycystic ovary syndrome (PCOS) and endocrine disrupting chemicals (EDCs). Rev Endocr Metab Disord.

[6] group TREA-sPcw (2004). Revised 2003 consensus on diagnostic criteria and long-term health risks related to polycystic ovary syndrome (PCOS). Hum Reprod.

[7] Subramaniam AG, Joseph A, Gupta A, Krishna MB, Laloraya M, Pillai SM (2015). Reduced Tregs in peripheral blood of PCOS patients – a consequence of aberrant Il2 signaling. J Clin Endocrinol Metab.

[8] Xiao N, He K, Gong F, Xie Q, Peng J, Su X (2019). Altered subsets and activities of B lymphocytes in polycystic ovary syndrome. J Allergy Clin Immunol.

[9] Garlanda C, Hirsch E, Bozza S, Salustri A, De Acetis M, Nota R (2002). Non-redundant role of the long pentraxin PTX3 in anti-fungal innate immune response. Nature..

[10] Camaioni A, Klinger FG, Campagnolo L, Salustri A (2018). The influence of pentraxin 3 on the ovarian function and its impact on fertility. Front Immunol.

[11] Varani S, Elvin JA, Yan C, DeMayo J, DeMayo FJ, Horton HF (2002). Knockout of pentraxin 3, a downstream target of growth differentiation factor-9, causes female subfertility. Mol Endocrinol.

[12] Tosi F, Di Sarra D, Bonin C, Zambotti F, Dall’Alda M, Fiers T (2014). Plasma levels of pentraxin-3, an inflammatory protein involved in fertility, are reduced in women with polycystic ovary syndrome. Eur J Endocrinol.

[13] Sahin FK, Sahin SB, Balik G, Ural UM, Tekin YB, Cure MC (2014). Does low pentraxin-3 levels associate with polycystic ovary syndrome and obesity?. Int J Clin Exp Med.

[14] Aydogdu A, Tasci I, Tapan S, Basaran Y, Aydogan U, Meric C (2012). High plasma level of long pentraxin 3 is associated with insulin resistance in women with polycystic ovary syndrome. Gynecol Endocrinol.

[15] Salustri A, Garlanda C, Hirsch E, De Acetis M, Maccagno A, Bottazzi B (2004). PTX3 plays a key role in the organization of the cumulus oophorus extracellular matrix and in in vivo fertilization. Development.

[16] McKenzie LJ, Pangas SA, Carson SA, Kovanci E, Cisneros P, Buster JE (2004). Human cumulus granulosa cell gene expression: a predictor of fertilization and embryo selection in women undergoing IVF. Hum Reprod.

[17] Wyskida K, Franik G, Choręza P, Pohl N, Markuszewski L, Owczarek A (2020). Pentraxin 3 levels in young women with and without polycystic ovary syndrome (PCOS) in relation to the nutritional status and systemic inflammation. Int J Endocrinol.

[18] Güdücü N, Görmüş U, Alp E, Kavak ZN (2014). Dünder İ. Reciprocal action of pentraxin-3 and Crp in women with polycystic ovary syndrome. European. J Inflamm.

[19] Herath S, Williams EJ, Lilly ST, Gilbert RO, Dobson H, Bryant CE (2007). Ovarian follicular cells have innate immune capabilities that modulate their endocrine function. Reproduction..

[20] Alessio P, Guido R, Andrea D, Somigliana FP, Liliana R (2006). Follicular fluid levels of the long pentraxin PTX3. J Soc Gynecol Investig.

[21] Salustri A, Yanagishita M, Underhill C, Laurent T, Hascall V (1992). Localization and synthesis of hyaluronic acid in the cumulus cells and mural granulosa cells of the preovulatory follicle. Dev Biol.

[22] Saltiel AR, Olefsky JM (2017). Inflammatory mechanisms linking obesity and metabolic disease. J Clin Invest.

[23] Bosutti A, Malaponte G, Zanetti M, Castellino P, Heer M, Guarnieri G (2008). Calorie restriction modulates inactivity-induced changes in the inflammatory markers C-reactive protein and pentraxin-3. J Clin Endocrinol Metab.

[24] Ogawa T, Kawano Y, Imamura T, Kawakita K, Sagara M, Matsuo T (2010). Reciprocal contribution of pentraxin 3 and C-reactive protein to obesity and metabolic syndrome. Obesity (Silver Spring).

[25] Osorio-Conles O, Guitart M, Chacón M, Maymo-Masip E, Moreno-Navarrete J, Montori-Grau M (2011). Plasma PTX3 protein levels inversely correlate with insulin secretion and obesity, whereas visceral adipose tissue PTX3 gene expression is increased in obesity. Am J Physiol Endocrinol Metab.

[26] Siu MK, Cheng CY. The blood-follicle barrier (BFB) in disease and in ovarian function. Biology and Regulation of Blood-Tissue Barriers: Springer; 2013. p. 186–92. 10.1007/978-1-4614-4711-5_9 PMC4169694 23397625

[27] Wang F, Pan J, Liu Y, Meng Q, Lv P, Qu F (2015). Alternative splicing of the androgen receptor in polycystic ovary syndrome. Proc Natl Acad Sci.

[28] Ibrahim A, Mutlu EC, Mehmet S, Hakan C, Mustafa O, Serkan T (2012). A macrophage activation marker chitotriosidase in women with PCOS: does low-grade chronic inflammation in PCOS relate to PCOS itself or obesity?. Arch Gynecol Obstet.

[29] Klein SL, Flanagan KL (2016). Sex differences in immune responses. Nat Rev Immunol.

[30] Ashcroft GS, Mills SJ (2002). Androgen receptor–mediated inhibition of cutaneous wound healing. J Clin Invest.

[31] Lee WJ, Jung HD, Chi SG, Kim BS, Lee S-J, Kim MK (2010). Effect of dihydrotestosterone on the upregulation of inflammatory cytokines in cultured sebocytes. Arch Dermatol Res.

[32] Kelly CCJ, Connell JMC, Lyall H, Gould GW, Sattar N, Petrie JR (2001). Low grade chronic inflammation in women with polycystic ovarian syndrome. J Clin Endocrinol Metab.

[33] Xiong Y-l, Liang X-y, Yang X, Li Y, Wei L-n (2011). Low-grade chronic inflammation in the peripheral blood and ovaries of women with polycystic ovarian syndrome. Eur J Obstet Gynecol Reprod Biol.

[34] Pan J-X, Zhang J-Y, Ke Z-H, Wang F-F, Barry JA, Hardiman PJ (2015). Androgens as double-edged swords: induction and suppression of follicular development. Hormones (Athens).

[35] González F, Sia CL, Bearson DM, Blair HE (2014). Hyperandrogenism induces a proinflammatory TNFα response to glucose ingestion in a receptor-dependent fashion. J Clin Endocrinol Metab.

[36] Li Y, Zheng Q, Sun D, Cui X, Chen S, Bulbul A (2019). Dehydroepiandrosterone stimulates inflammation and impairs ovarian functions of polycystic ovary syndrome. J Cell Physiol.

[37] Yang J, Zhang C, Li L, Huang L, Ji S, Lu C (2010). Testosterone induces redistribution of forkhead box-3a and down-regulation of growth and differentiation factor 9 messenger ribonucleic acid expression at early stage of mouse folliculogenesis. Endocrinology.

